# Diabetes in Patients With Pancreatic Neuroendocrine Neoplasms

**DOI:** 10.3389/fendo.2020.615082

**Published:** 2020-12-23

**Authors:** Xiaoling Zhuge, Yajie Wang, Xiao Chen, Chuangen Guo

**Affiliations:** ^1^ Department of Laboratory Medicine, The First Affiliated Hospital, Zhejiang University School of Medicine, Hangzhou, China; ^2^ Department of Radiology, The Affiliated Hospital of Nanjing University of Chinese Medicine, Nanjing, China; ^3^ Department of Radiology, The First Affiliated Hospital, Zhejiang University School of Medicine, Hangzhou, China

**Keywords:** diabetes mellitus, pancreatic neuroendocrine neoplasms, high-density lipoprotein, fasting plasma glucose, tumor grade

## Abstract

**Objective:**

Diabetes mellitus (DM) is probably a risk factor for pancreatic neuroendocrine neoplasms (PNENs). However, the prevalence of DM in PNEN patients remains inconclusive. In the present study we observed the prevalence of DM and possible risk factors in PNEN patients.

**Methods:**

After excluding those with insulinoma, a total of 197 patients with PNENs were included. The demographic data, pathological characteristics, and data of blood biochemical tests were recorded. DM was considered if there was evidence of a fasting plasma glucose level of ≥7.0 mmol/L or a 2-h plasma glucose level of ≥11.1 mmol/L, or a history of DM at the time of PNEN diagnosis. Impaired fasting glucose was considered if fasting plasma glucose level was between 6.1 and 7.0 mmol/L.

**Results:**

The prevalence of DM, new-onset DM, and impaired fasting glucose were 17.26, 9.14, and 7.1%, respectively. The prevalence of DM was 26.0% in patients ≥60 years old (19/73) and 12.1% in patients <60 years old. Multivariable logistic regression analysis demonstrated that age, tumor size, and nerve invasion were independent risk factors for DM and impaired fasting glucose + DM (p < 0.05). Age, organs and nerve invasion were independent risk factors for impaired fasting glucose. Low high-density lipoprotein (HDL) was also a risk factor for incident of DM (OR = 0.15, 95%CI: 0.03–0.66). G2/G3 was an independent risk factor for DM in women.

**Conclusion:**

Our data shows that the prevalence of DM is 17.26% in patients with PNENs and is 26.0% in patients ≥60 years of age after excluding insulinoma. Age, nerve invasion, tumor size, and HDL are risk factors for DM in PNEN patients.

## Introduction

Neuroendocrine neoplasms (NENs) are a group of uncommon lesions that usually occur in the gastroenteropancreatic (GEP) tract or in the bronchopulmonary system (1). The pancreas is one of the most commonly affected organs in NENs (PNENs). PNENs account for 1–2% of pancreatic tumors (2, 3). However, recent studies show that the incidence of PNENs is increasing due to the development in diagnostic techniques (2–4).

The pancreas is also the critical organ for glucose metabolism, as it secretes insulin. Several studies have shown the association between diabetes mellitus (DM) and incident of PNENs (5–8). Those data indicate that DM is probably a risk factor for PNEN occurrences. PNENs may also affect glucose metabolism. Tumor cells may secrete hormones that can affect glucose metabolism or insulin resistance. In addition, tumor mass may influence normal insulin secretion by inducing destruction or atrophy of pancreatic parenchyma (7, 9, 10).

DM has become a challenge for public health in China (11). Few studies have shown the prevalence of DM or impaired fasting glucose in PNENs (5, 12, 13). Ben et al. (5) indicated that the prevalence of T2DM was 16.9% in a Chinese PNEN population. However, poorly differentiated neuroendocrine carcinomas were excluded from that study. A recent study in China demonstrated that the prevalence of type 2 DM (T2DM) was 20.2% (12). In addition, Fan et al. showed that T2DM may be related to the biological behavior of PNENs, such as distant metastases and nerve invasion (12). Insulinoma was included in those two Chinese studies. A recent study showed that 28.3% of PNENs (insulinoma or glucagonoma was excluded) patients had DM or dysglycemia (blood glucose >140 mg/dl) in a German population (13). Insulinoma can decrease glucose levels by excessively secreting insulin and cause hypoglycemia. It would be better to exclude insulinoma from those studies. In addition, the prevalence of DM in PNEN patients is different between studies. Thus, we speculated that the prevalence of DM in PNENs remains inconclusive, and that further study is required. Anti-diabetic drugs may be a potential strategy for adjuvant therapy of PNENs (14, 15). It would be valuable to know the prevalence of DM or impaired fasting glucose in PNEN patients for treatment planning. In the present study we aimed to show the prevalence of DM in PNEN patients after excluding insulinoma and identify possible associated factors.

## Materials and Methods

### Study Population

This cross-sectional study was approved by the Ethics Board of the First Affiliated Hospital, College of Medicine, Zhejiang University. Informed consent was waived because of the retrospective design. We searched our medical record from January 2011 to May 2020. We found a total of 242 surgically or cytologically proven PNEN patients who did not receive medical treatment for their tumor. Subjects with missing information, such as tumor grade, glucose data or medical history, and with a history of malignant disease and chronic pancreatitis were excluded from the final analysis (n = 16). In addition, patients with insulinoma that decreased glucose levels (n = 29) by excessively secreting insulin and type 1 DM (n = 0) were not included in this study. Finally, 197 PNEN patients were included. We recorded the demographic data and data of blood biochemical tests.

### Blood Biochemical Tests

Blood biochemical tests included fasting plasma glucose levels, 2-h plasma glucose levels, serum total cholesterol (TC), serum triglyceride (TG), high-density lipoprotein (HDL), and low-density lipoprotein (LDL). All blood biochemical tests were performed within one week before the operation.

### Definition of DM

Definition of DM was based on plasma glucose levels and history of DM. DM was considered if the fasting plasma glucose level was ≥7.0 mmol/L or the 2-h plasma glucose level was ≥11.1 mmol/L during the oral glucose tolerance test. Moreover, DM was considered if a history of DM occurred at the time of PNEN diagnosis. Impaired fasting glucose was considered if the fasting plasma glucose level was between 6.1 mmol/L and 7.0 mmol/L. Patients with normal glucose levels or with no history of DM were regarded as non-diabetic patients. The glucose level was determined within one week before the operation. New-onset diabetes mellitus was defined as DM diagnosed within 2–3 years before a PNEN diagnosis (16).

### Histology of PNENs

We recorded the following histological PNEN data: tumor size, location, ki67 index, mitotic count, lymph node invasion, organs invasion, vascular invasion, and nerve invasion. The tumor grades were defined based on the 2017 WHO classification for NENs (17). Grade 1(G1): mitosis count was <2/10 HPF and/or Ki-67 ≤2; Grade 2 (G2): mitosis count was 2–20/10 HPF and/or Ki-67 index was 3–20; Grade 3 (G3): mitosis count was >20 per 10 HPF, Ki-67 index was >20%. G3 neoplasm was not divided into well-differentiated G3 and pancreatic neuroendocrine carcinoma (PNEC) because the separation depended on the genetic backgrounds (p53 and *KRAS* mutations) of the two groups (18) that were not obtained in our population.

### Statistical Analysis

Statistical analysis was performed using commercial software SPSS 16.0 (SPSS Inc, Chicago, IL). Data was shown as mean ± standard deviation or number of cases (percentage). Independent sample t test or Mann–Whitney U-test was used for continuous data and Chi-square test or Fisher’s exact test was used for categorical variables. Univariable and multivariable logistic regression analyses were used to show the association between high fasting glucose level or DM and demographic data (age, BMI and gender), histological data of PNENs (ki67 index, mitotic count, lymph node invasion, organs invasion, vascular and nerve invasion), tumor size, and location or data of blood biochemical tests (HDL, LDL, TG, and TC). Statistical significance was considered if P value <0.05.

## Results

### Characteristic of Patients

Characteristics of patients are listed in **Table 1**. There were 93 women and 104 men. The average age was 56.06 years old. The average size was 3.37 cm and ki67 index was 15.2. There were 66 PNEN G1, 85 G2, and 46 G3. The prevalence of DM, impaired fasting glucose, and new-onset DM were 17.26, 7.11, and 9.14%, respectively. Further, 52.9% of DM was new-onset DM. No6 significant differences were found in the prevalence of DM, new-onset DM and impaired fasting glucose between men and women. The prevalence of DM was 26.0% in patients ≥60 years old (19/73) and 12.1% in patients <60 years old. The G3 PNENs occurred more commonly in men than in women (p = 0.01). Consequently, the ki67 index and vascular invasion in men was higher than in women (p < 0.05 and p < 0.01).

**Table 1:** Characteristic of patients.

Variables	Total (n = 197)	Women (n = 93)	Men (n = 104)	p
Age (years)	56.06 ± 11.65	55.82 ± 10.99	56.27 ± 12.27	0.78
Sex	93/104	/	/	
Size (cm)	3.37 ± 2.27	3.36 ± 2.07	3.39 ± 2.45	0.93
ki67 (%)	15.2 ± 23.56	10.04 ± 18.78	19.88 ± 26.06	<0.01
Glucose (mmol/L)	5.53 ± 1.84	5.61 ± 2.15	5.47 ± 1.51	0.63
TG (mmol/L)	1.38 ± 0.84	1.41 ± 0.88	1.35 ± 0.82	0.57
TC (mmol/L)	4.34 ± 1.06	4.43 ± 1.12	4.27 ± 1.00	0.28
HDL (mmol/L)	1.14 ± 0.37	1.19 ± 0.38	1.11 ± 0.35	0.14
LDL (mmol/L)	2.45 ± 0.81	2.50 ± 0.90	2.40 ± 0.73	0.40
Grade (1/2/3)	66/85/46	35/45/13	31/40/33	0.01
Location				0.43
Head-neck	93	46	47	
Body	66	27	39	
Tail	38	23	15	
Diabetes mellitus (yes)	34 (17.26%)	17 (18.28%)	17 (16.34%)	0.72
New-onset diabetes mellitus	18 (9.14%)	10 (10.75%)	8 (7.69%)	0.46
Impaired fasting glucose (yes)	14 (7.11%)	9 (9.68%)	5 (4.81%)	0.18
Impaired fasting glucose + Diabetes mellitus	48 (24.37%)	27 (29.03%)	21 (20.19%)	0.15
Duration of DM (years)*	0.6 (0–4.0)	2.0 (0–4.0)	0.0 (0–5.5)	0.97
Lymph node invasion	18 (9.14%)	9 (9.68%)	9 (8.65%)	0.87
Organs invasion	29 (14.72%)	12 (12.90%)	17 (16.35%)	0.50
Vascular invasion	27 (13.71%)	8 (8.60%)	19 (18.27%)	0.04
Nerve invasion	20 (10.15%)	7 (7.53%)	13 (12.50%)	0.25

TC, Serum total cholesterol; TG, serum triglyceride; HDL, high-density lipoprotein; LDL, low-density lipoprotein.

*Data was shown as median (interquartile range).

Subsequently, we showed the characteristic of patients based on impaired fasting glucose (**Table 2**) or DM (**Table 3**). The age of patients with impaired fasting glucose or DM was older than those that had none. The sizes of tumors in DM patient were bigger than those that had none (p = 0.03). A chi-square for trend analysis showed that the prevalence of DM was increased with tumor grades (p = 0.10). No such trends were observed in patients with impaired fasting glucose. Organ invasions were more common in PNENs that had impaired fasting glucose than that had none (p < 0.01). Nerve invasion was more common in PNENs that had DM than with none (p < 0.01).

**Table 2:** Characteristic of patients based on impaired fasting glucose.

Variables	Impaired fasting glucose (n = 14)	Non-Impaired fasting glucose (n = 149)	p
Age (years)	60.53 ± 9.50	54.53 ± 12.00	0.06
Length (cm)	3.18 ± 1.78	3.21 ± 2.00	0.95
ki67(%)	19.67 ± 31.85	14.01 ± 21.82	0.46
Glucose (mmol/L)	6.47 ± 0.19	4.90 ± 0.58	<0.01
TG (mmol/L)	1.48 ± 0.54	1.33 ± 0.88	0.54
TC (mmol/L)	4.84 ± 1.17	4.28 ± 0.97	0.04
LDL (mmol/L)	2.88 ± 1.00	2.39 ± 0.74	0.02
HDL(mmol/L)	1.25 ± 0.43	1.17 ± 0.36	0.43
BMI(kg/m^2^)	24.1 ± 4.3	23.6 ± 3.8	0.34
Grade (1/2/3)*	5/6/3	54/64/31	0.99
Location*			0.59
Head-neck	8	67	
Body	3	51	
Tail	3	31	
Lymph node invasion	3	11	0.11
Organs invasion	6	15	<0.01
Vascular invasion	1	21	0.55
Nerve invasion	2	10	0.28

*Chi-square for trend.

TC, Serum total cholesterol; TG, serum triglyceride; HDL, high-density lipoprotein; LDL, low-density lipoprotein.

**Table 3:** Characteristic of patients with and without diabetes mellitus.

Variables	Diabetes mellitus (n = 34)	Non-Diabetes mellitus (n = 163)	p
Age (years)	60.24 ± 8.75	55.06 ± 11.97	0.02
Sex (F/M)	17/17	85/91	0.62
Length (cm)	4.14 ± 3.31	3.22 ± 1.97	0.03
ki67(%)	16.53 ± 23.60	14.92 ± 23.37	0.26
Glucose (mmol/L)	9.33 ± 3.35	5.05 ± 0.73	<0.01
TG (mmol/L)	1.51 ± 0.80	1.35 ± 0.85	0.20
TC (mmol/L)	4.25 ± 1.13	4.36 ± 1.05	0.76
HDL (mmol/L)	2.41 ± 0.92	2.45 ± 0.79	0.90
HDL(mmol/L)	0.98 ± 0.33	1.17 ± 0.36	0.01
BMI (kg/m^2^)	24.9 ± 4.6	24.0 ± 4.8	0.16
Grade (1/2/3)*	8/15/11	58/70/35	0.10
Location*			0.39
Head-neck	17	76	
Body	13	53	
Tail	4	34	
Lymph node invasion	4	14	0.54
Organs invasion	8	21	0.11
Vascular invasion	6	21	0.40
Nerve invasion	8	12	<0.01
Hypoglycemic agents			
Insulin	4		
Sulfonylureas	7		
Metformin	5		
Glucosidase inhibitor	2		
Other	1		

*Chi-square for trend

TC, Serum total cholesterol; TG, serum triglyceride; HDL, high-density lipoprotein; LDL, low-density lipoprotein.

### The Glucose Levels

**Figure 1:**
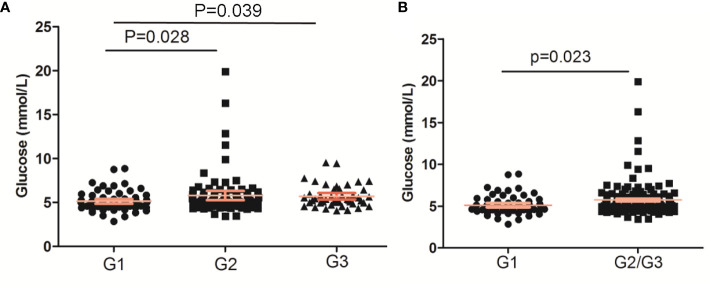
The glucose levels based on WHO grade of pancreatic neuroendocrine neoplasms. **(A)** PNENs were divided into three groups; **(B)** PNENs were divided into two groups.

The glucose levels in PNENs G2 and G3 were both higher than that in PNEN G1 (p < 0.05) (**Figure 1A**). A similar result was found between G1 and G2/G3 (p = 0.023) (**Figure 1B**). The prevalence of impaired fasting glucose and DM in G2/G3 tumor was also higher than those of G1 tumors (27.4 *vs* 18.2% and 19.85 *vs* 12.12%), but no significant differences were found.

### Associated Factors With Impaired Fasting Glucose and DM

Finally, we adopted a logistic regression analysis to show the potential associated factors of impaired fasting glucose and DM (**Table 4**). Univariable analysis showed that age, organs, TC, and LDL were associated with incidents of impaired fasting glucose (all p < 0.05). Moreover, age, size, nerve invasion, HDL, and G3 were associated with DM. Yet still, age, organs and nerve invasions were associated with incident of impaired fasting glucose + DM (all p < 0.05). Multivariable analysis demonstrated that age, tumor size and nerve invasion were independent associated factors for DM and impaired fasting glucose + DM (p < 0.05). Low HDL was an associated factor for DM (OR = 0.15, 95%CI: 0.03–0.66). Age, organ invasion, and nerve invasion were independent associated factors for impaired fasting glucose (p < 0.05).

**Table 4:** Logistic regression analysis in total population.

	Impaired fasting glucose*		Diabetes mellitus		Impaired fasting glucose + Diabetes mellitus	
	Univariable	Multivariable	Univariable	Multivariable	Univariable	Multivariable
Variables	OR (95%CI)	OR (95%CI)	OR (95%CI)	OR (95%CI)	OR (95%CI)	OR (95%CI)
Age (years)	1.06(1.00–1.12)	1.09(1.01–1.18)	1.05(1.01–1.09)	1.06(1.02–1.11)	1.04(1.01–1.08)	1.06(1.02–1.11)
Sex (Male *vs* female)	0.45(0.15–1.42)	0.55(0.13–2.42)	0.87(0.42–1.83)	0.62(0.27–1.45)	0.53(0.27–1.06)	0.51(0.23–1.10)
Tumor size (cm)	0.98(0.73–1.30)	0.97(0.60–1.58)	1.17(1.01–1.35)	1.22(1.00–1.49)	1.13(0.99–1.30)	1.19(1.00–1.44)
Organs invasion (yes *vs* no)	6.70(2.05–21.92)	15.24(2.23–104.1)	2.08(0.84-5.20)	1.16(0.40–3.41)	3.68(1.62–8.35)	2.53(0.95–6.77)
Nerve invasion (yes *vs* no)	2.31(0.45–11.81)	17.28(1.37–218.1)	3.87 (1.44–10.39)	3.05 (1.01–9.60)	2.90(1.12–7.49)	3.51(1.11–11.14)
TG (mmol/L)	1.23(0.73–2.10)	1.81(0.66–4.94)	1.22(0.82–1.80)	1.03(0.56–1.90)	1.27(0.89–1.81)	1.13(0.66–1.99)
TC (mmol/L)	1.78(1.05–3.02)	1.69(0.89–2.86)	1.00(0.71–1.42)	0.64(0.28–1.50)	1.24(0.92–1.68)	0.69(0.29–1.64)
HDL (mmol/L)	1.61(0.37–7.07)	3.56(0.19–65.3)	0.20(0.06–0.61)	0.15(0.03–0.66)	0.44(0.17–1.11)	0.36(0.10–1.31)
LDL (mmol/L)	2.19(1.14–4.20)	13.94(0. 35–551.2)	1.00(0.63–1.57)	2.10(0.75–5.88)	1.35(0.91–2.01)	2.44(0.88–6.80)
Grade						
G1	1	1	1	1	1	1
G2	1.01(0.29–3.50)	0.39(0.05–3.00)	1.81(0.69–4.73)	1.27(0.44–3.67)	1.48(0.67–3.27)	0.72(0.27–1.93)
G3	1.05(0.23–4.67)	0.18(0.01–2.91)	2.98(1.07–8.28)	1.68(0.50–5.67)	2.18(0.91–5.24)	0.72(0.21–2.45)
Location						
Head-neck	1	1	1	1	1	1
Body	0.49(0.12–1.95)	0.31(0.05–1.86)	1.10(0.50–2.45)	1.26(0.50–3.16)	0.76(0.36–1.58)	0.76(0.33–1.79)
Tail	0.81(0.20–3.27)	0.52(0.09–3.25)	0.53(0.17–1.68)	0.61(0.18–2.11)	0.58(0.23–1.49)	0.51(0.18–1.47)

TC, Serum total cholesterol; TG, serum triglyceride; HDL, high-density lipoprotein; LDL, low-density lipoprotein.

Multivariable regression analysis was additionally adjusted with vascular and lymph node invasion.

*Patients with diabetes mellitus were not included.

For the female population, we found that age, HDL, and G3 were independent associated factors for DM after adjustment with potential confounders. OR was 1.17 (95%CI: 1.07–1.30), 0.07 (95%CI: 0.07–1.00), and 10.13 (95%CI: 1.30–78.94), respectively.

## Discussion

Several case–control studies showed that DM was a risk factor for PNENs (6, 8). However, few data has been reported on the prevalence of DM in PNENs. Capurso et al. (6) showed 10 cases of recent-onset diabetes and 17 cases of DM in 162 PNENs, which indicated a prevalence of 16.7% in the Italian population. Recently, Fan et al. (12) and Ben et al. (5) indicated that the prevalence of DM was 20.2 and 16.9% in two Chinese populations, respectively. However, insulinoma which may cause hypoglycemia is included in the three studies, wherein poorly differentiated neuroendocrine carcinomas were excluded from Fan’s study. In the present study insulinoma was excluded and the prevalence of DM and recent-onset diabetes was 17.26 and 9.14%. For patients older than 60 years of age, the prevalence of DM was 26.0%. We also observed that age, tumor size, nerve invasion and HDL levels were independent associated factors for DM in PNEN patients.

The prevalence of DM in China has been reported in several investigations (11, 19). The prevalence of diabetes was 11.5–12.9% and 20.2–20.4% among subjects who were 40 to 59 and ≥60 years of age, respectively. In our study we found that the prevalence of diabetes was 12.1% in PNEN patients <60 years of age, which was similar to the national survey data. However, our data showed that the prevalence of diabetes in PNEN patients ≥60 years (26.0%) was higher than the estimated prevalence of diabetes patients in China. The prevalence of diabetes increases with increasing age (11, 19). Similar data was also found in PNEN patients. We found that age was an independent associated factor of DM in PNEN patients. Atrophy of pancreatic parenchyma is common in old persons. We speculated that the tumor effects of PNENs on pancreatic parenchyma may be more severer in older people. Moreover, we also found that the incident of DM increased when tumor size increased. Gallo et al. (7) reported that the PNEN tumor size in patients with diabetes was greater than those that did not have diabetes. Large tumor mass may induce more severe destruction or atrophy of pancreatic parenchyma (7, 20). However, tumor size was not considered in Fan’s study (12).

DM and PNENs may have bidirectional associations (7). DM may be a risk factor for PNEN occurrence (21). However, the specific mechanism linking DM to PNENs has not been clarified. DM-related chronic inflammation and oxidative stress may play an important role (5). Some PNEN secret hormones can induce hyperglycemia and insulin resistance (21), such as glucagonomas or somatostatinomas. In addition, direct tumor effects of PNENs may cause the obstruction of the pancreatic duct and promote atrophy of pancreatic parenchyma, ultimately affecting insulin secretion (10, 21). Surgical treatment or drug therapy, such as pancreatectomy and somatostatin analogs may also affect glucose metabolism (7). Those subjects in our study did not receive any PNEN-related medical therapy before operations.

A few studies have shown the role of DM in the biological behavior of PNENs (12). The risks of nerve invasion and distant metastasis were higher in PNEN patients with diabetes or dysglycemia than those without diabetes (12, 13). Our study found that nerve invasion was associated with impaired fasting glucose and DM, which was consistent with previous studies. Diabetes can induce damage to peripheral nerves (22). Is the damage related to nerve invasion? The mechanism of nerve invasion on occurrence of DM or DM on nerve invasion in PNENs needed further study. In addition, our data showed that impaired fasting glucose was associated with organ invasion. Fan et al. (12) showed that the risk of DM in PNEN patients with distant metastases was higher than without it. A similar association was also observed in PNEN patients with dysglycemia (blood glucose >140 mg/dl) (13). Previous data also indicated that PNENs’ grade was associated with DM (12). However, the number of G3 PNENs in Fan’s study was small (n = 11). We did not find the association between DM or impaired fasting glucose and PNEN’s grade. We speculated that those negative results may be due to the exclusion of insulinoma from our study. Insulinoma usually showed low grade G1 or G2 (23) and low risk of organ invasion. Interestingly, we found that the higher tumor grade was significantly associated with DM in the female population which was consistent with a recent study (24). There may be gender differences of PNENs in terms of associated comorbidities (24). Our data also supports that G3 PNENs are more common in male patients (18).

Lipid abnormalities, such as high TG and a low concentration of HDL, are also associated with DM (25). However, effects of lipid abnormalities on DM have not been considered in previous studies (12). Our results indicated that low HDL level was also an independent risk factor for DM in PNEN patients.

The present study has several limitations. First, some factors related to development of DM, such as alcohol consumption, cigarette smoking, nutrition, and physical activity were not considered. Second, the data came from a single institution, which weakened generalizability of the results. Third, the tumor stage was not included in the logistic regression analysis. Fourth, the diagnosis of DM was not based on HbA1C because HbA1C was not routinely determined in our institution. Finally, the small sample size might have led to the observed, null associations between some variables and DM.

In conclusion, our data showed that the prevalence of DM and new-onset DM were 17.26 and 8.63% after excluding patients with insulinoma. For patients older than 60 years, the prevalence of DM was 26.0%. The prevalence of DM in PNENs was higher than that of the general Chinese adult population. In addition, we found that age, tumor size, nerve invasion, and HDL levels were potential risk factors for DM in PNEN patients.

## Data Availability Statement

The original contributions presented in the study are included in the article/supplementary material. Further inquiries can be directed to the corresponding authors.

## Ethics Statement

The studies involving human participants were reviewed and approved by the Ethics Board of the First Affiliated Hospital, Zhejiang University School of Medicine. Written informed consent for participation was not required for this study in accordance with the national legislation and the institutional requirements.

## Author Contributions

XC and CG contributed to conception of the study. XZ, YW, and XC contributed to interpretation of data and preparation of the manuscript. XC and CG performed the data analysis and contributed to the discussion. All authors contributed to the article and approved the submitted version.

## Funding

This study was funded by Zhejiang Provincial Natural Science Foundation (LY18H180004) and Zhejiang Medical Science and Technology Project (2017KY331). A Project Supported by Scientific Research Fund of Zhejiang Provincial Education Department (Y202043424).

## Conflict of Interest

The authors declare that the research was conducted in the absence of any commercial or financial relationships that could be construed as a potential conflict of interest.
